# Exploring the impact of body mass index on tumor biology and cancer development

**DOI:** 10.1007/s00432-024-05890-4

**Published:** 2024-07-27

**Authors:** Johanne Ahrenfeldt, Stine Carstensen, Ida Maria Hemdorff Eriksen, Nicolai Juul Birkbak

**Affiliations:** 1 Department of Molecular Medicine, Aarhus University Hospital, Aarhus, Denmark; 2 Department of Clinical Medicine, Aarhus University, Aarhus, Denmark; 3 Bioinformatics Research Center, Aarhus University, Aarhus, Denmark

**Keywords:** Cancer and obesity, Transcriptomics, Tumor aggresiveness, Pathway analysis, Tumor biology

## Abstract

**Purpose:**

Cancer continues to be a major global health challenge, affecting millions of individuals and placing substantial burdens on healthcare systems worldwide. Recent research suggests a complex relationship between obesity and cancer, with obesity increasing the risk of various cancers while potentially improving outcomes for diagnosed patients, a phenomenon termed the "obesity paradox". In this study, we used a cohort of 1781 patients to investigate the impact of obesity on tumor characteristics, including gene expression, pathway dysfunction, genetic alterations and immune infiltration.

**Methods:**

Patient samples spanned 10 different cancer types, and were obtained from the Cancer Genome Atlas, with annotations for body mass index (BMI), age, sex, tumor size and tumor gene expression data.

**Results:**

When we compared the proportion of large (T3–T4) to small tumors (T1–T2) between obese and non-obese patients, we found that obese patients tended to present with smaller, less invasive tumors and exhibited distinct gene expression profiles, particularly in metabolic and proliferative pathways. Moreover, smaller tumors in obese patients show higher immune cell infiltration and increased T cell diversity, suggesting enhanced immune activity.

**Conclusion:**

Taken together, these findings highlight the influence of obesity on tumor biology, with implications for personalized treatment strategies that consider patient physiology alongside tumor characteristics.

**Supplementary Information:**

The online version contains supplementary material available at 10.1007/s00432-024-05890-4.

## Introduction

Cancer, characterized by uncontrolled cell growth and proliferation, remains a significant global health concern, posing substantial challenges to both healthcare systems and individuals. Understanding the multifaceted factors influencing cancer development is crucial for devising effective prevention and intervention strategies. One emerging area of research centers around the association between obesity and cancer. Obesity, resulting from an imbalance between energy intake and expenditure, has reached epidemic proportions worldwide. In 2016, 650 million people in the world were characterized as obese according to the World Health Organization (WHO). Beyond its established role in metabolic disorders, evidence has been mounting on the potential link between obesity and cancer incidence (Renehan et al. 2008; Ma et al. 2013; Genkinger et al. 2011; Wallin and Larsson 2011; Sanfilippo et al. 2014; Wang and Xu 2014). Numerous epidemiological and metastudies have suggested that obesity is associated with an increased risk of several cancer types, including breast, colorectal, and renal cancers (Renehan et al. 2008; Wang and Xu 2014; Islami et al. 2019; Thrift et al. 2014) While the exact mechanisms underlying this association are still under investigation, chronic inflammation, altered hormonal profiles, and insulin resistance are among the proposed pathways through which obesity may contribute to tumorigenesis (Roberts et al. 2010; Gallagher and LeRoith 2015; Liu et al. 2021).

Despite the established link between obesity and increased cancer risk, an intriguing phenomenon known as the “obesity paradox" has been observed across cancer types (Schlesinger et al. 2014; Hakimi et al. 2013; Amptoulach et al. 2015). Paradoxically, although obesity can increase the risk of developing certain cancers, several studies suggest that obese individuals with cancer may have better outcomes than their non-obese counterparts. The reasons for this phenomenon are not fully understood, but it is speculated that obese individuals might be more resilient to treatment, as they experience less severe chemotherapy-induced toxicity (Tsang et al. 2016; Cay et al. 2024) or that their physiology might induce less aggressive cancer metabolic profiles (Wang et al. 2019).

In the context of cancer aggressiveness, there is a growing interest in elucidating whether obesity influences the development of cancer with distinct phenotypic characteristics. While previous work has predominantly focused on the association between obesity and overall cancer risk, investigating the specific impact of obesity on specific cancer subtypes, potentially those exhibiting reduced aggressiveness, is essential for a more nuanced understanding of the impact of obesity on cancer development and outcome. Addressing this knowledge gap is critical not only for discerning the molecular and cellular underpinnings of obesity-related carcinogenesis, but also for tailoring treatment strategies to specific phenotypic features of obesity-induced cancer. By exploring whether obesity plays a role in the development of aggressive cancer phenotypes, it may open up novel avenues for cancer prevention and treatment within an increasing population of obese individuals.

One of the important hallmarks of cancer is immune evasion (Hanahan and Weinberg 2011), as the immune system also works as a defense against development of cancer. The cancer cells must develop mechanisms to avoid the immune system in order to survive. An important part of the immune system's defense against cancer are cytotoxic T cells, capable of recognizing and killing cancer cells. T cells harbor the T-cell receptor (TCR), which recognizes mutation-induced neo-antigens produced by the cancer cells. A recent study suggests that a greater TCR diversity in the tumor is associated with a highly activated tumor microenvironment (Schina et al. 2023).

With this study, we used gene expression data obtained from 10,783 patients from the Cancer Genome Atlas to investigate if tumors from obese patients displayed phenotypic variation relative to tumors from non-obese patients. Across cancer types, we observed that tumors from obese patients were significantly smaller at diagnosis, and showed significantly altered gene expression patterns, particularly affecting genes in metabolic and proliferative pathways. Furthermore, through analysis of T cell receptor diversity, we infer likely variation in immunological profiles between tumors from obese and non-obese individuals. Overall, our work demonstrates that obesity itself significantly impacts not only the risk of developing cancer, but also the type of cancer, with likely implications for patient treatment decisions and prognosis.

## Methods

### Data

Clinical information from 10,783 sequenced tumor samples from 33 different cancer types was acquired from The Cancer Genome Atlas (Ellrott et al. 2018). RNAseq-based gene expression data which had been uniformly normalized for all samples was acquired from the University of California Santa Cruz (UCSC) Xena database (Goldman et al. 2020). Pathological T-stage was used as a measurement for tumor size and invasiveness. After omitting missing values from the following variables: T-stage, age, and sex; the data set contained 7309 cancer patients and 23 cancer types, this will be referred to as Subset 1. Of these, a subset of 1781 cancer patients from 10 cancer types were annotated with BMI values, when excluding extreme outlier values (BMI below 15 or above 60), this will be referred to as Subset 2. Additionally, T cell Receptor (TCR) diversity was available for 5,366 patients, obtained from Thorsson et al. (Thorsson et al. 2018).

### Gene sets and immune cell decomposition

Gene set variation analysis (GSVA) (Hänzelmann et al. 2013) was performed to generate values for 50 Hallmark pathways from Liberzon et al. (Liberzon et al. 2015) from the gene expression data.

Tumor immune cell decomposition was calculated as the tumor infiltrating leukocytes (TIL) score defined by Danaher et al (Danaher et al. 2017) on whole tumor RNAseq data using the method described in Rosenthal et al (Rosenthal et al. 2019).

### Enrichment analysis

For the enrichment analysis we looked at cancer driver mutations. Mutations were annotated as driver events using Annovar (Wang et al. 2010) as previously described in Ahrenfeldt et al (Ahrenfeldt et al. 2022). Briefly we used PolyPhen (Ng and Henikoff 2001) and SIFT (Adzhubei et al. 2010) to predict if mutations were deleterious, likely resulting in a loss of function in tumor suppressor genes, or pathogenic in oncogenes. Enrichment analysis was performed using a two-sided Fisher exact test to compare large tumors to small tumors, on the number of patients with and without altered genes, per cancer type. The P values were corrected by false discovery rate (FDR) and a corrected P value below 0.05 was considered significant.

The driver weight was calculated for each patient as 1/number of driver mutations, and then we calculated the mean difference in driver weight between small and large tumors per gene per cancer type. The P value for each gene-cancertype pair was calculated using a Wilcoxon rank sum test, and then corrected using FDR, a corrected P value below 0.05 was considered significant.

### Statistical analysis

All data analysis was performed in R version 4.3.0 (R Core Team 2020), using tidyverse (Wickham et al. 2019), survminer (Kassambara et al. 2021), survival (Therneau and Grambsch 2000), scales (Hadley and Seidel 2019), ggpubr (Kassambara 2020), ggAU-package (Kisistok J ggAU: ggplot2 themes for Aarhus University.Preprint at(2023) 2023) and Publish (Gerds and Ozenne 2021).

Survival analyses were performed by Cox proportional hazard regression (Cox 1972) and Kaplan meier curves.

Testing the significance of differences between groups was performed using the Wilcoxon rank sum test, unless otherwise mentioned. Fisher's exact test was used to determine if the proportion of small tumors was higher in a subset of the data. A binomial test was performed to test whether the distribution of cancer types which were significantly higher expressed in small and large tumors for each hallmark was significantly different from 50/50. All p-values are two-sided.

## Results

### Patients and samples

**Fig. 1:**
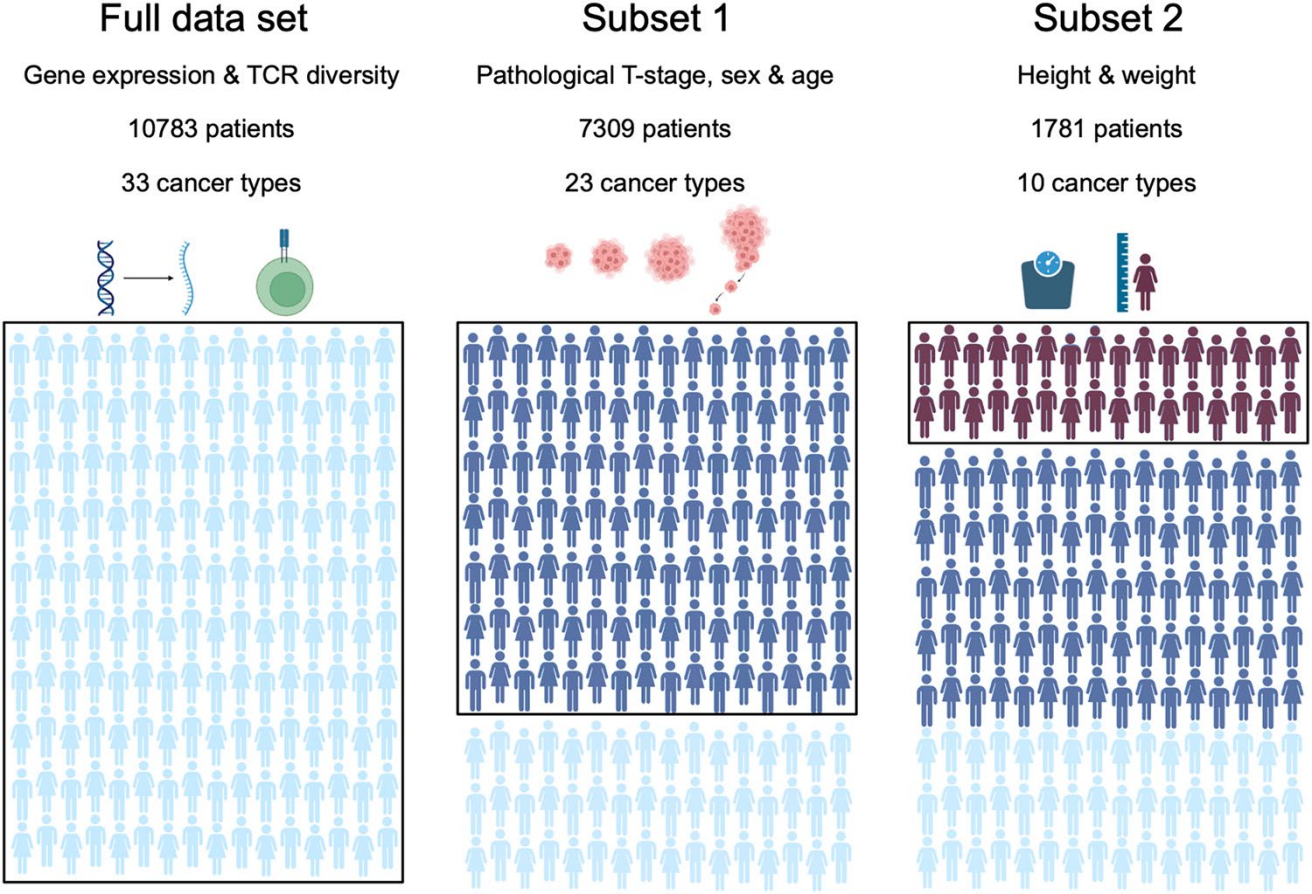
Data cohort. A schematic representation of the full data set from TCGA and Thorsson et al*.* 2018, and the two subsets that we perform the analysis on. For the full data set we have gene expression data and T-cell receptor diversity information. For Subset 1, which includes 7309 of the patients from the full data set, we have pathological T-stage, age and sex annotations for all patients. For Subset 2, which includes 1781 patients from Subset 1, we have height and weight information for all patients at diagnosis. The figure was created using BioRender

To investigate the association between obesity and tumor aggression and size, we performed transcriptional pathway analysis and statistical analysis on data from The Cancer Genome Atlas (TCGA). From the full data set with 10,783, we defined two nested subsets of data. Subset 1 consisted of 7309 patients spanning 23 different cancer types, all annotated with information on age, sex, and pathological tumor stage (T-stage). Subset 2 consisted of 1781 patients, all from Subset 1, who had Body Mass Index (BMI) information available, these patients spanned 10 cancer types (Fig. 1).

### Patients with high BMI more commonly harbor smaller, less invasive tumors

**Fig. 2:**
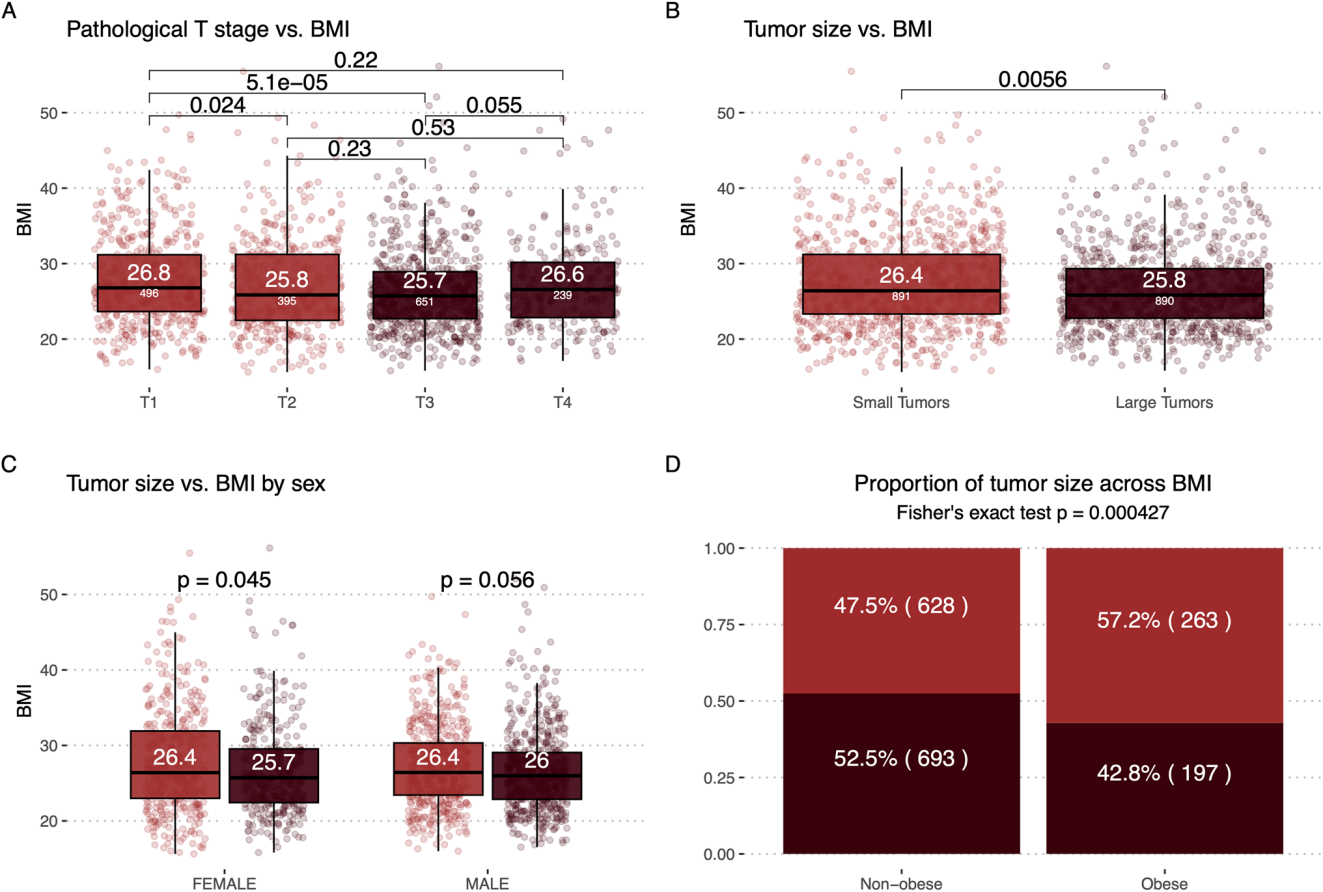
BMI and pathological T-stage on Subset 2. **A** Patients BMI stratified by their tumor’s pathological T stage. Colored by tumor size (Small: T1 and T2, Large: T3 and T4). **B** Patients BMI plotted stratified by their tumor size. **C** Patients BMI plotted against their tumor size stratified by sex. **D** Patients are stratified by obesity, BMI >  = 30, and the proportion of small and large tumors are shown for each group

First, we endeavored to investigate if obese individuals in general present with smaller tumors, indicative of a less aggressive phenotype driving early cancer development. To explore this, we used pathological T stage as a proxy for tumor size. T stage is a component of the standardized TNM (Tumor, Node, Metastasis) staging system developed by the American Joint Committee on Cancer (AJCC) and used globally for staging cancers (Edge and Compton 2010). As part of this, T stage describes the size and extent of the primary tumor, and is typically graded as T1-T4. While the exact definition varies by cancer type, T1 tumors are typically smaller, while T4 tumors are larger and may have more extensive growth into local tissue. When we compared the BMI of patients based on T stage, we found that patients with low T stage, particularly T1 tumors, had higher BMI relative to patients with higher stage tumors (Fig. 2A). When we stratified the patients into two groups based on T stage, small tumors (T1, T2) and large tumors (T3, T4), we found that patients with small tumors had a significantly higher BMI (median = 26.4), relative to patients with large tumors (median = 25.8, P = 0.0056) (Fig. 2B). There was no significant difference in BMI by sex in this cohort (female median = 26, male median 26.2, P = 0.68). However, we observed the same pattern within each sex, where patients with small tumors had a significantly higher BMI relative to patients diagnosed with larger tumors (small tumors, female median = 26.4, male median 26.4; large tumors, female median = 25.7, male median 26, P female = 0.045, P male = 0.056, Fig. 2C). When we further stratified patients based on BMI into obese (BMI >  = 30) and non-obese (BMI < 30), we found a significant enrichment of small tumors in patients with obesity (Obese 57.2% vs Non-obese 47.5%, P = 0.000427) (Fig. 2d).

### Small tumors in patients with high BMI show unique immune profiles

**Fig. 3:**
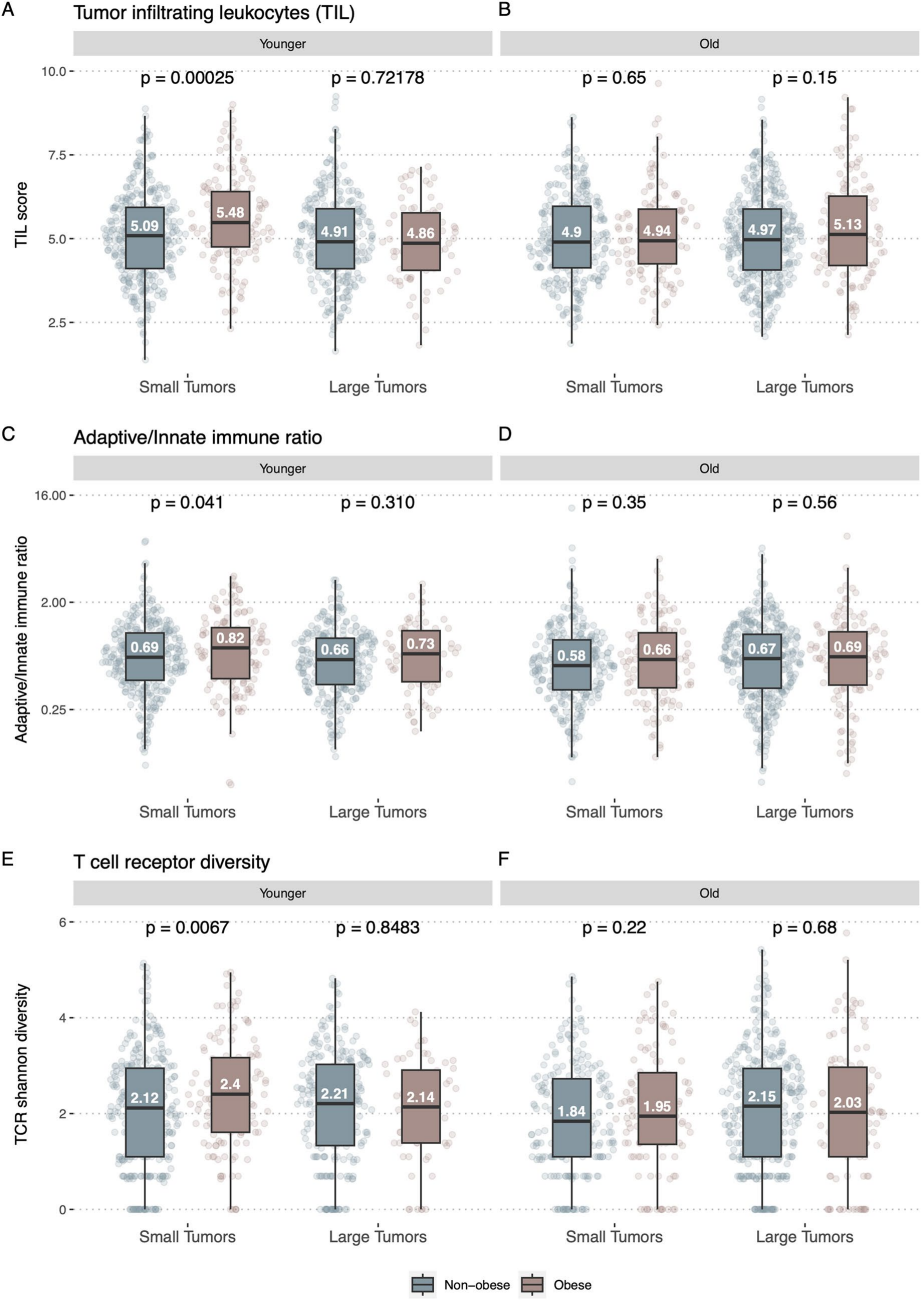
Tumor immune infiltration and diversity. **A** Tumor Infiltrating leukocytes (TIL) score in the younger (< 60 years) patients. The patients are stratified by tumor size and colored by obesity (non-obese: BMI < 30, obese: BMI >  = 30). **B** TIL score in the older (> = 60 years) patients. The patients are stratified by tumor size and colored by obesity. **C** Adaptive/innate immune ratio of younger patients. The patients are stratified by tumor size and colored by obesity. The Y-axis is log2-scaled. **D** Adaptive/innate immune ratio of older patients. The patients are stratified by tumor size and colored by obesity. The Y-axis is log2-scaled. **E** T-cell receptor (TCR) shannon diversity of younger patients. The patients are stratified by tumor size and colored by obesity. **F** TCR shannon diversity of older patients. The patients are stratified by tumor size and colored by obesity

To investigate if smaller tumors from obese patients may be the result of more aggressive immune activity, we explored the differences in immune cell infiltration between small and large tumors from obese and non-obese patients. Given that the immune system decays with age due to immunosenescence (Pawelec 2018), we further stratified these analyses based on age. We investigated immune infiltration by utilizing the TIL score from Danaher (Danaher et al. 2017), and found that the small tumors of obese patients had a significantly higher level of immune infiltration relative to their non-obese counterparts (P = 0.00025), in younger (< 60 years) patients (Fig. 3A). We observed no differences in immune infiltration within older patients nor between the larger tumors in patients with or without obesity (Fig. 3B).

Next, we investigated the composition of infiltrating immune cells using the ratio of adaptive to innate immune cells (A/I ratio). We have previously shown that within tumors the A/I ratio is associated with improved survival (Ahrenfeldt et al. 2023). Here, we found that in younger patients with small tumors, obese patients had a higher A/I ratio relative to non-obese patients (P = 0.041) (Fig. 3C). We found no significant differences in the older patients (Fig. 3D).

To investigate the landscape of tumor infiltrating adaptive immune cells, we obtained TCR diversity and richness estimates from the TCGA data, previously published by Thorsson et al. (Thorsson et al. 2018). We found that small tumors exhibited a significantly higher TCR Shannon diversity index in younger patients with obesity relative to younger patients without obesity (P = 0.0067) (Fig. 3E). We found no significant difference in the older cohort with small tumors or between obese and non-obese patients with large tumors, neither in the young nor old cohort (Fig. 3F).

### Tumors from obese individuals show distinct pathway expression profiles

**Fig. 4:**
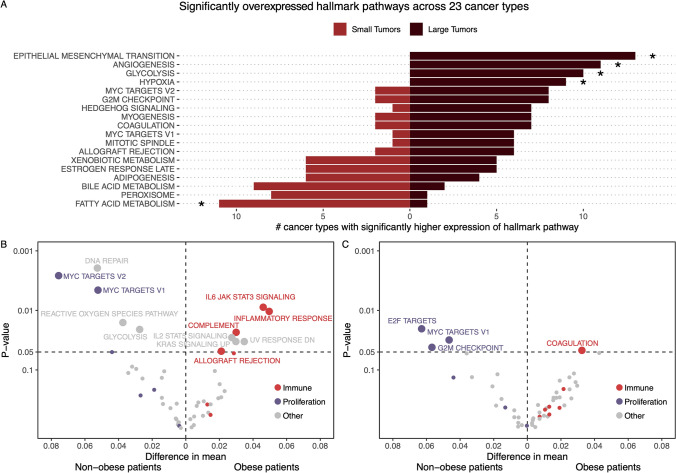
Differences in pathway expression in small and large tumors. **A** A bar plot showing the pathways where there are more than 5 cancer types with an overexpression in small or large tumors, and the number of cancer types that are significantly overexpressed in either direction. An asterisk, *, marks the pathways where the distribution of cancer types into small or large are significantly different from 50/50, given a binomial distribution. This analysis is performed on Subset 1. **B** A volcano plot showing the difference of mean (and p-value given a t-test) GSVA values for each pathway between non-obese and obese patients with small tumors. The pathways are colored by their overall process category. This analysis is performed on Subset 2. **C** A volcano plot showing the difference of mean (and p-value given a t-test) GSVA values for each pathway between non-obese and obese patients with large tumors. The pathways are colored by their overall process category. This analysis is performed on Subset 2

Tumor size is strongly prognostic, and is therefore likely associated with a more aggressive biological phenotype. To investigate this, we compared gene expression profiles between small and large tumors across the 7309 samples from 23 cancer types in Subset 1 with T stage annotations, and compared large tumors to small tumors within each cancer type. For this analysis, we summarized gene expression to pathways, gene set variation analysis (GSVA) of the 50 hallmark pathways (Liberzon et al. 2015). All pathways were tested for significant differential expression across all 23 cancer types. In this manner, we observed that 38 showed a significantly different expression between small and large tumors at least once, ranging from 0 to 15 significant pathways per cancer type (Fig S1A). To summarize these results across cancer types, the hallmark pathways were scored as either significantly expressed or not significantly expressed in each cancer type, using an FDR adjusted p-value of 0.1 as cutoff. We then used a binomial test to determine if a hallmark pathway was significantly enriched across multiple cancer types. Here, we found that large tumors have a significantly higher expression of the EPITHELIAL_MESENCHYMAL_TRANSITION, ANGIOGENESIS, and HYPOXIA pathways, all of which have previously been associated with poor outcome and aggressive cancer (Thiery et al. 2009; Oshi et al. 2021; Evans and Koch 2003). Furthermore, we found large tumors to have a significantly higher expression of the GLYCOLYSIS metabolic pathway, whereas small tumors have a significantly higher expression of FATTY_ACID_METABOLISM (Fig. 4A). We also found that proliferative pathways such as MYC_TARGETS_V1 and V2 and G2M_CHECKPOINT were most highly expressed in large tumors, although this was not significant.

Next, to investigate the impact of obesity in tumor phenotype, we further explored if obesity might impact the observed differences between small and large tumors in Subset 1. By comparing gene expression data between obese and non-obese patients, within small and large tumors separately, we observe lower expression of the proliferative pathways (small tumors: MYC_TARGETS_V1 and V2, large tumors: E2F_TARGETS, MYC_TARGETS_V1 and G2M_CHECKPOINT) and higher expression of immune related pathways (small tumors: IL6_JAK_STAT3_SIGNALING, INFLAMMATORY_RESPONSE, COMPLEMENT and ALLOGRAFT_REJECTION, large tumors: COAGULATION) in both small (Fig. 4B) and large tumors (Fig. 4C) in obese patients.

To investigate the cancer-specific origin of the differential expression, we stratified the analysis on cancer type and found that for small tumors, overexpression of the proliferative pathways in non-obese patients were predominantly driven by liver cancer, esophagus cancer and renal cancer (Fig S1B). Likewise, overexpression of immune pathways in obese patients mostly originated from liver cancer and bladder cancer. In large tumors overexpression of the proliferative pathways in non-obese patients mostly originated from liver cancer and colon cancer, while overexpression of immune pathways in obese patients mostly originated from melanoma and uveal melanoma (Fig S1C).

To investigate if there were differences in gene expression between older and younger patients, we performed the analysis stratified into older and younger patients, as above. We found that when we compared RNA expression from small tumors between younger and older patients, tumors from younger patients had a higher expression of proliferative pathways, such as E2F_TARGETS, G2M_CHECKPOINT and MITOTIC_SPINDLE. Conversely, in small tumors from older patients we found a higher expression of metabolic pathways including XENOBIOTIC_METABOLISM, BILE_ACID_METABOLISM, FATTY_ACID_METABOLISM, HEME_METABOLISM and OXIDATIVE_PHOSPHORYLATION (Fig S2A). When we repeated the analysis in large tumors, we found that younger patients had a higher expression of TGF_BETA_SIGNALING and APICAL_JUNCTION while no pathways had a significantly higher expression in older patients (Fig S2B).

Next, to investigate if there were any significant differences in the expression between the two sexes, we performed the same analysis stratified by sex. For this analysis we excluded sex-specific cancer types, BRCA, CESC, PRAD and TGCT. When we compared small tumors between male and female patients, we found no significant difference (Fig S3A). When we performed the analysis using large tumors, we found that 18 of the 50 pathways are significantly higher expressed in female patients compared to male patients (Fig S3B), these include mainly immune related pathways (INFLAMMATORY_RESPONSE, COMPLEMENT, IL6_JAK3_STAT_SIGNALING, ALLOGRAFT_REJECTION, INTERFERON_GAMMA_RESPONSE and COAGULATION) and signaling pathways (TNFA_SIGNALING_VIA_NKFB, IL2_STAT5_SIGNALING, KRAS_SIGNALING, ESTROGEN_REPONSE_EARLY and ESTROGEN_RESPONSE_LATE).

### Genotypic patterns in large vs small tumors

**Fig. 5:**
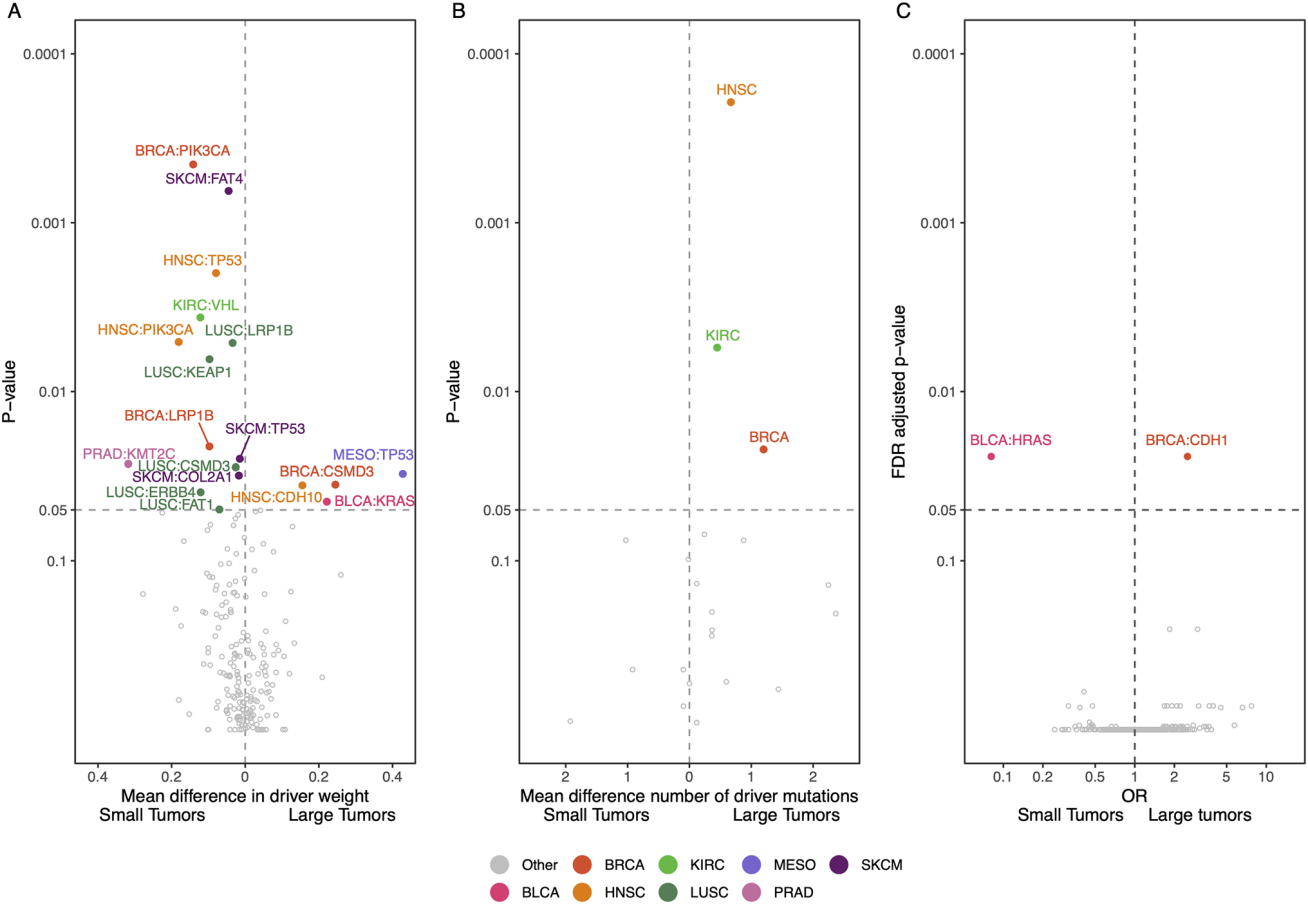
Driver genes and tumor size. **A** A volcano plot showing the mean difference of driver weight per gene, per cancer type between small and large tumors. The driver weight is 1/number of driver mutations per tumor. The p-value is calculated by a t-test. **B** A volcano plot showing the mean difference in number of driver mutations per tumor for each cancer type between small and large tumors. The p-value is calculated by a t-test. **C** A volcano plot showing the odds ratio for an enrichment of certain driver mutations in small or large tumors. Odd ratio and p-value is calculated by fisher’s exact test

To investigate if the landscape of cancer driver mutations might differ between small and large tumors, we categorized all mutations found within known cancer genes in tumors from Subset 1 into whether they were likely driver mutations or likely passenger mutations. We explored how often individual cancer driver mutations occurred together with other driver mutations within the same tumor. To investigate this, we defined a driver weight score. The driver weight score was determined for each driver mutation, within each tumor, as simply 1/n_driver_. We then compared the differences in mean driver weight across genes and cancer types. We found that there were more genes with a significantly higher driver weight in small tumors relative to in large tumors (Fig. 5A). Examples of these are PIK3CA in both BRCA and HNSC, LRP1B in both LUSC and BRCA and TP53 in HNSC and SKCM. However, TP53 also has a higher driver weight in Large MESO tumors. To investigate whether small tumors had a higher driver weight in general, we compared the driver weight of small vs large tumors for each cancer type, where the tumor's driver weight was the same as for each of its driver mutations 1/n_driver_. We investigated the mean difference in driver mutations between small and large tumors and found that in three cancer types (BRCA, HNSC and KIRC) large tumors had significantly higher number of driver mutations (Fig. 5B). When we looked at the frequency of specific driver mutations between large and small tumors we only found two significantly enrichment genes (Fig. 5C), HRAS in small BLCA tumors and CDH1 in large BRCA tumors.

## Discussion

Our study suggests a link between obesity and reduced tumor size as we found a significantly higher BMI in the patients with smaller, less invasive tumors, as represented by lower T stage. We also found an association between obesity and increased immune invasion and lower expression of proliferative pathways, suggesting that tumors in obese individuals may harbor less aggressive biology. Our results thus support previous work indicating that while the obesity induced chronic inflammatory state may support tumorigenesis, it may also limit tumor growth through immune effector mechanisms (Multhoff et al. 2011).

Furthermore, we found an increased expression of metabolic pathways, including the fatty acid and bile acid pathway in small tumors and in obese patients. We found an increased expression of the glycolysis pathway in the large tumors. This may indicate that small tumors grow on fatty acid, whereas larger tumors preferentially utilize glucose, via glycolysis and then lactic acid fermentation, i.e. the Warburg effect (Warburg 1925). It is possible that tumors develop to preferentially metabolize fatty acids due to a more plentiful supply of free fatty acids in the plasma of obese patients (Henderson 2021).

Previous studies have also found a high level of variation between tumors and the tumor microenvironment between men and women (Ahrenfeldt et al. 2023). However we do not find this difference between men and women, when we stratify based on BMI. Here, we found no difference in BMI between male and female cancer patients in the analyzed cohort. We also found the same distribution of BMI of patients with small or large tumors in male and female patients. Furthermore, when we investigated the differentially expressed pathways between male and female patients in small or large tumors, few differences were found. This indicates that the differential expression pattern that we found in small tumors in patients with obesity, was independent of sex.

In our study, we found that the main genetic difference between small and large tumors was the number of driver mutations, as we found fewer driver mutations per tumor in small tumors. And when we investigate if specific mutations were enriched in small or large tumors, we found only two genes, HRAS in small bladder cancer tumors and CDH1 in large breast cancer tumors. This indicates that on a genomic level, there is no difference between the molecular drivers of cancer between small and large tumors. These results thus follow the pattern of previous research, where we and others have found that there is no significant difference between the cancer driver landscape between primary and metastatic tumors (Ahrenfeldt et al. 2022; Christensen et al. 2022).

Taken together, we here demonstrate that obesity may affect tumor biology, our findings are thus important in the context of personalized medicine. We show an effect of the host physiology on both tumor microenvironment and molecular characteristics, thus providing a more nuanced understanding of how obesity might affect cancer development. Our work thus highlights the limits of a tumor-centric approach to tumor characterization, where patient prognosis and treatment is primarily determined from single tumor biopsies. Rather, these results indicate that a holistic approach is needed, where overall patient characteristics are considered in order to properly determine optimal patient care.

### Supplementary Information

Below is the link to the electronic supplementary material.

Supplementary file1 (PDF 595 KB)

## Data Availability

No datasets were generated or analysed during the current study.

## References

[CR1] Adzhubei IA et al (2010) A method and server for predicting damaging missense mutations. Nat Methods 7:248–249 20354512 10.1038/nmeth0410-248 PMC2855889

[CR2] Ahrenfeldt J et al (2022) Computational analysis reveals the temporal acquisition of pathway alterations during the evolution of cancer. Cancers 14:5817 36497297 10.3390/cancers14235817 PMC9739002

[CR3] Ahrenfeldt J et al (2023) The ratio of adaptive to innate immune cells differs between genders and associates with improved prognosis and response to immunotherapy. PLoS ONE 18:e0281375 36745657 10.1371/journal.pone.0281375 PMC9901741

[CR4] Amptoulach S, Gross G, Kalaitzakis E (2015) Differential impact of obesity and diabetes mellitus on survival after liver resection for colorectal cancer metastases. J Surg Res 199:378–385 26115811 10.1016/j.jss.2015.05.059

[CR5] Cay G et al (2024) Harnessing physical activity monitoring and digital biomarkers of frailty from pendant based wearables to predict chemotherapy resilience in veterans with cancer. Sci Rep 14:2612 38297103 10.1038/s41598-024-53025-z PMC10831115

[CR6] Christensen DS et al (2022) Treatment represents a key driver of metastatic cancer evolution. Cancer Res 82:2918–2927 35731928 10.1158/0008-5472.CAN-22-0562

[CR7] Cox DR (1972) Regression models and life-tables. J R Stat Soc 34:187–202 10.1111/j.2517-6161.1972.tb00899.x

[CR8] Danaher P et al (2017) Gene expression markers of tumor Infiltrating Leukocytes. J Immunother Cancer 5:18 28239471 10.1186/s40425-017-0215-8 PMC5319024

[CR9] Edge SB, Compton CC (2010) The American Joint Committee on Cancer: the 7th edition of the AJCC cancer staging manual and the future of TNM. Ann Surg Oncol 17:1471–1474 20180029 10.1245/s10434-010-0985-4

[CR10] Ellrott K et al (2018) Scalable open science approach for mutation calling of tumor exomes using multiple genomic pipelines. Cell Syst 6:271-281.e7 29596782 10.1016/j.cels.2018.03.002 PMC6075717

[CR11] Evans SM, Koch CJ (2003) Prognostic significance of tumor oxygenation in humans. Cancer Lett 195:1–16 12767506 10.1016/S0304-3835(03)00012-0

[CR12] Gallagher EJ, LeRoith D (2015) Obesity and diabetes: the increased risk of cancer and cancer-related mortality. Physiol Rev 95:727–748 26084689 10.1152/physrev.00030.2014 PMC4491542

[CR13] Genkinger JM et al (2011) A pooled analysis of 14 cohort studies of anthropometric factors and pancreatic cancer risk. Int J Cancer 129:1708–1717 21105029 10.1002/ijc.25794 PMC3073156

[CR14] Gerds TA, Ozenne B (2021) Publish: Format Output of Various Routines in a Suitable Way for Reports and Publication. Preprint at https://CRAN.R-project.org/package=Publish

[CR15] Goldman MJ et al (2020) Visualizing and interpreting cancer genomics data via the Xena platform. Nat Biotechnol 38:675–678 32444850 10.1038/s41587-020-0546-8 PMC7386072

[CR16] Hadley W, Seidel D (2019) Scales: scale functions for visualization. Preprint at https://CRAN.R-project.org/package=scales

[CR17] Hakimi AA et al (2013) An epidemiologic and genomic investigation into the obesity paradox in renal cell carcinoma. J Natl Cancer Inst 105:1862–1870 24285872 10.1093/jnci/djt310 PMC3866155

[CR18] Hanahan D, Weinberg RA (2011) Hallmarks of cancer: the next generation. Cell 144:646–674 21376230 10.1016/j.cell.2011.02.013

[CR19] Hänzelmann S, Castelo R, Guinney J (2013) GSVA: gene set variation analysis for microarray and RNA-seq data. BMC Bioinform 14:7 10.1186/1471-2105-14-7 PMC3618321 23323831

[CR20] Henderson GC (2021) Plasma free fatty acid concentration as a modifiable risk factor for metabolic disease. Nutrients 13:2590 34444750 10.3390/nu13082590 PMC8402049

[CR21] Islami F, Goding Sauer A, Gapstur SM, Jemal A (2019) Proportion of cancer cases attributable to excess body weight by US State, 2011–2015. JAMA Oncol 5:384–392 30589925 10.1001/jamaoncol.2018.5639 PMC6521676

[CR22] Kassambara A (2020) ggpubr: ‘ggplot2’ Based Publication Ready Plots. Preprint at https://rpkgs.datanovia.com/ggpubr/

[CR23] Kassambara A, Kosinski M, Biecek P (2021) Survminer: Drawing Survival Curves using ‘ggplot2’. Preprint at https://CRAN.R-project.org/package=survminer

[CR24] Kisistok J ggAU: ggplot2 themes for Aarhus University. Preprint at (2023)

[CR25] Liberzon A et al (2015) The molecular signatures database (MSigDB) hallmark gene set collection. Cell Syst 1:417–425 26771021 10.1016/j.cels.2015.12.004 PMC4707969

[CR26] Liu X-Z, Pedersen L, Halberg N (2021) Cellular mechanisms linking cancers to obesity. Cell Stress Chaperones 5:55–72 10.15698/cst2021.05.248 PMC8090860 33987528

[CR27] Ma Y et al (2013) Obesity and risk of colorectal cancer: a systematic review of prospective studies. PLoS ONE 8:e53916 23349764 10.1371/journal.pone.0053916 PMC3547959

[CR28] Multhoff G, Molls M, Radons J (2011) Chronic inflammation in cancer development. Front Immunol 2:98 22566887 10.3389/fimmu.2011.00098 PMC3342348

[CR29] Ng PC, Henikoff S (2001) Predicting deleterious amino acid substitutions. Genome Res 11:863–874 11337480 10.1101/gr.176601 PMC311071

[CR30] Oshi M et al (2021) Angiogenesis is associated with an attenuated tumor microenvironment, aggressive biology, and worse survival in gastric cancer patients. Am J Cancer Res 11:1659–1671 33948380 PMC8085878

[CR31] Pawelec G (2018) Age and immunity: what is ‘immunosenescence’? Exp Gerontol 105:4–9 29111233 10.1016/j.exger.2017.10.024

[CR32] R Core Team. R: A Language and Environment for Statistical Computing. Preprint at https://www.R-project.org/ (2020).

[CR33] Renehan AG, Tyson M, Egger M, Heller RF, Zwahlen M (2008) Body-mass index and incidence of cancer: a systematic review and meta-analysis of prospective observational studies. Lancet 371:569–578 18280327 10.1016/S0140-6736(08)60269-X

[CR34] Roberts DL, Dive C, Renehan AG (2010) Biological mechanisms linking obesity and cancer risk: new perspectives. Annu Rev Med 61:301–316 19824817 10.1146/annurev.med.080708.082713

[CR35] Rosenthal R et al (2019) Neoantigen-directed immune escape in lung cancer evolution. Nature 567:479–485 30894752 10.1038/s41586-019-1032-7 PMC6954100

[CR36] Sanfilippo KM et al (2014) Hypertension and obesity and the risk of kidney cancer in 2 large cohorts of US men and women. Hypertension 63:934–941 24637660 10.1161/HYPERTENSIONAHA.113.02953 PMC4098147

[CR37] Schina A et al (2023) Intratumoral T-cell and B-cell receptor architecture associates with distinct immune tumor microenvironment features and clinical outcomes of anti-PD-1/L1 immunotherapy. J Immunother Cancer 11:e006941 37604641 10.1136/jitc-2023-006941 PMC10445359

[CR38] Schlesinger S et al (2014) Postdiagnosis body mass index and risk of mortality in colorectal cancer survivors: a prospective study and meta-analysis. Cancer Causes Control 25:1407–1418 25037235 10.1007/s10552-014-0435-x

[CR39] Therneau TM, Grambsch PM (2000) Modeling survival data: extending the COx model. Springer New York, NY, New York

[CR40] Thiery JP, Acloque H, Huang RYJ, Nieto MA (2009) Epithelial-mesenchymal transitions in development and disease. Cell 139:871–890 19945376 10.1016/j.cell.2009.11.007

[CR41] Thorsson V et al (2018) The immune landscape of cancer. Immunity 48:812-830.e14 29628290 10.1016/j.immuni.2018.03.023 PMC5982584

[CR42] Thrift AP et al (2014) Obesity and risk of esophageal adenocarcinoma and Barrett’s esophagus: a Mendelian randomization study. J Natl Cancer Inst 106:dju252 25269698 10.1093/jnci/dju252 PMC4200028

[CR43] Tsang NM et al (2016) Overweight and obesity predict better overall survival rates in cancer patients with distant metastases. Cancer Med 5:665–675 26811258 10.1002/cam4.634 PMC4831285

[CR44] Wallin A, Larsson SC (2011) Body mass index and risk of multiple myeloma: a meta-analysis of prospective studies. Eur J Cancer 47:1606–1615 21354783 10.1016/j.ejca.2011.01.020

[CR45] Wang F, Xu Y (2014) Body mass index and risk of renal cell cancer: a dose-response meta-analysis of published cohort studies. Int J Cancer 135:1673–1686 24615287 10.1002/ijc.28813

[CR46] Wang K, Li M, Hakonarson H (2010) ANNOVAR: functional annotation of genetic variants from high-throughput sequencing data. Nucleic Acids Res 38:e164 20601685 10.1093/nar/gkq603 PMC2938201

[CR47] Wang Z et al (2019) Paradoxical effects of obesity on T cell function during tumor progression and PD-1 checkpoint blockade. Nat Med 25:141–151 30420753 10.1038/s41591-018-0221-5 PMC6324991

[CR48] Warburg O (1925) The metabolism of carcinoma cells. J Cancer Res 9:148–163 10.1158/jcr.1925.148

[CR49] Wickham H et al (2019) Welcome to the tidyverse. J Open Source Softw 4:1686 10.21105/joss.01686

