# Supplementary material for: Exploring the impact of body mass index on tumor biology and cancer development
Source: J Cancer Res Clin Oncol. 2024 Jul 27;150(7):372. doi: 10.1007/s00432-024-05890-4 (PMC11283407; doi:10.1007/s00432-024-05890-4)
Supplement: Supplementary file 1 — Supplementary file1 (PDF 595 KB) [file 432_2024_5890_MOESM1_ESM.pdf]

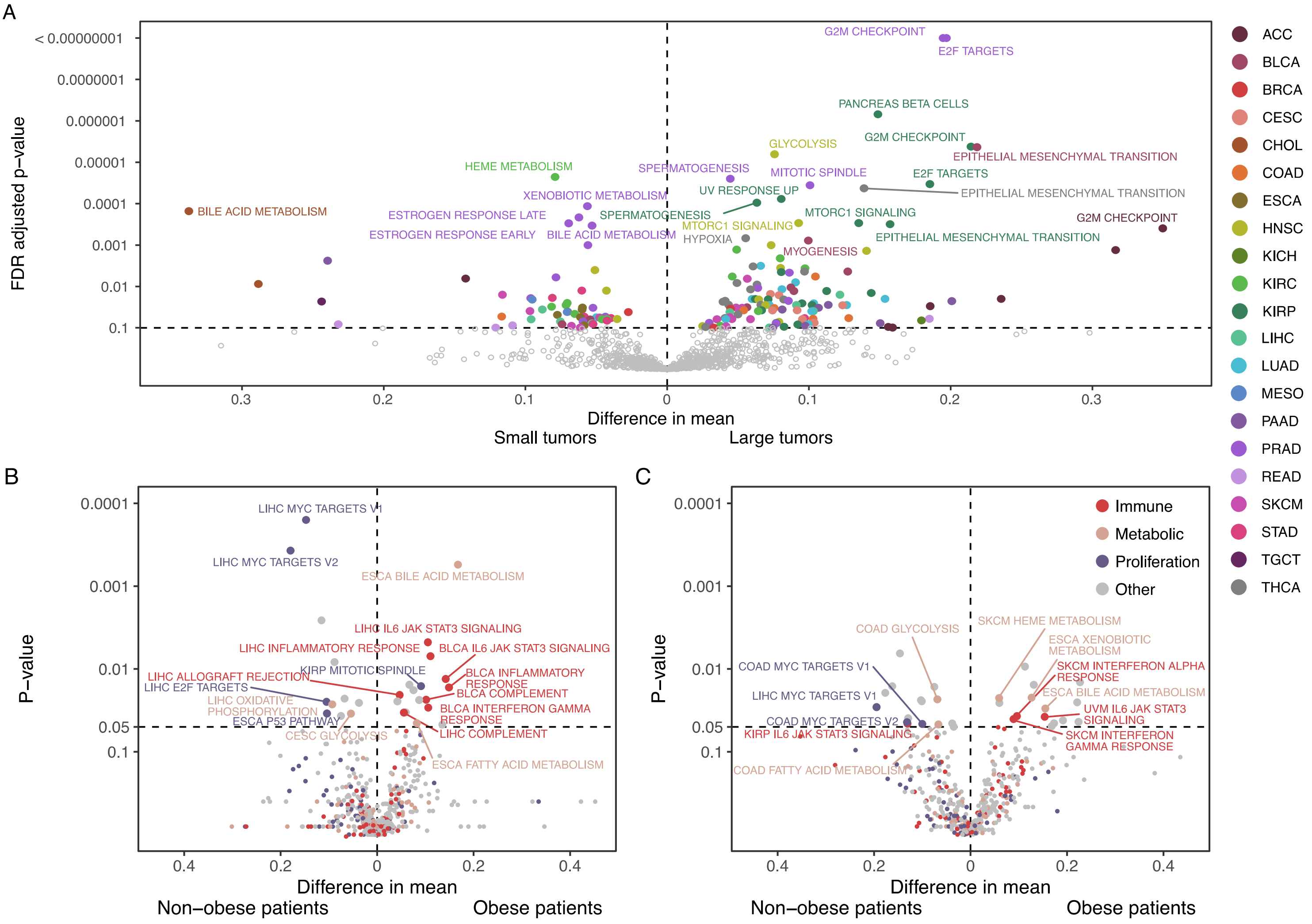

Supplementary Figure 1. *A) a volcano plot showing the difference in mean GSVA values between small and large tumors for the 50 hallmarks for each of the 23 cancer types in Subset 1. B) A volcano plot showing the difference of mean (and p-value given a t-test) GSVA values for each pathway between non-obese and obese patients with small tumors for each of the 10 cancer types in Subset 2. The pathways are colored by their overall process category. C) A volcano plot showing the difference of mean (and p-value given a t-test) GSVA values for each pathway between non-obese and obese patients with large tumors for each of the 10 cancer types in Subset 2. The pathways are colored by their overall process category.*

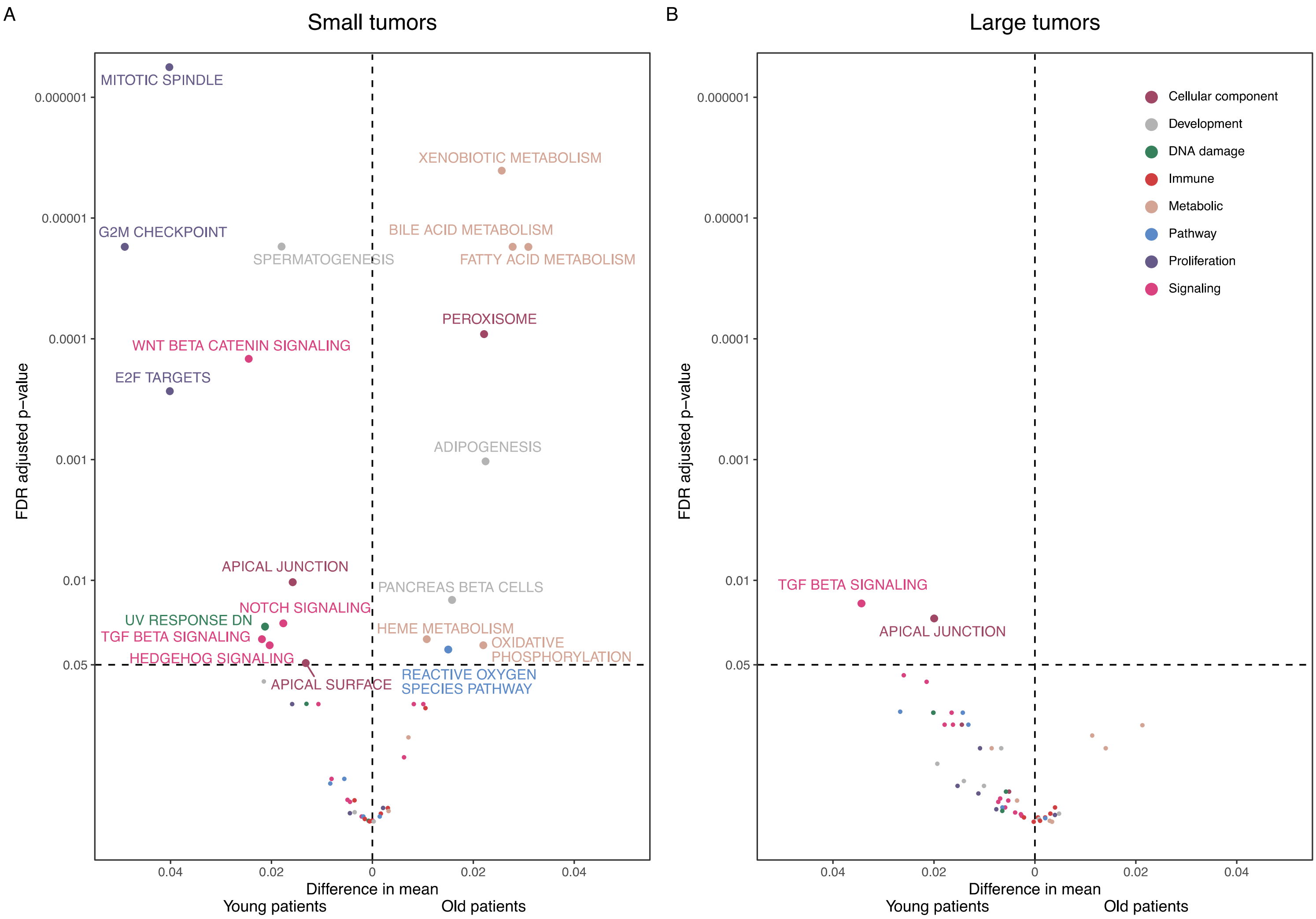

Supplementary Figure 2. *A) A volcano plot showing the difference of mean (and p-value given a t-test) GSVA values for each pathway between young and old patients with small tumors in Subset 1. The pathways are colored by their overall process category. B) A volcano plot showing the difference of mean (and p-value given a t-test) GSVA values for each pathway between young and old patients with large tumors in Subset 1. The pathways are colored by their overall process category.*

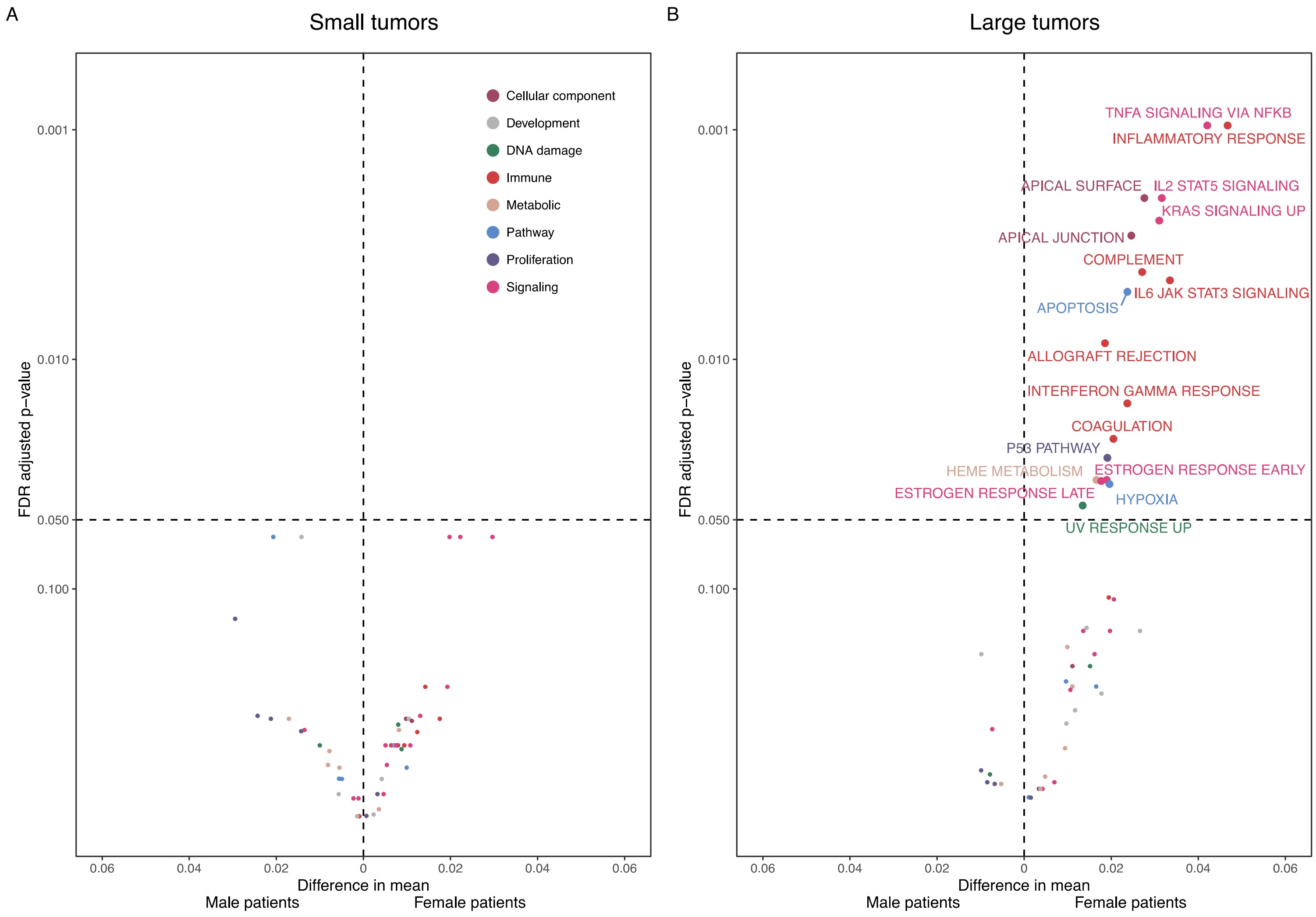

Supplementary Figure 3. A) A volcano plot showing the difference of mean (and p-value given a t-test) GSVA values for each pathway between male and female patients with small tumors in Subset 1. The pathways are colored by their overall process category. B) A volcano plot showing the difference of mean (and p-value given a t-test) GSVA values for each pathway between male and female patients with large tumors in Subset 1. The pathways are colored by their overall process category.
